# Establishing Reference Intervals for Bone Turnover Markers in the Healthy Shanghai Population and the Relationship with Bone Mineral Density in Postmenopausal Women

**DOI:** 10.1155/2013/513925

**Published:** 2013-02-27

**Authors:** Wei-Wei Hu, Zeng Zhang, Jin-Wei He, Wen-Zhen Fu, Chun Wang, Hao Zhang, Hua Yue, Jie-Mei Gu, Zhen-Lin Zhang

**Affiliations:** Metabolic Bone Disease and Genetic Research Unit, Department of Osteoporosis and Bone Disease, Shanghai Sixth People's Hospital Shanghai Jiao Tong University, 600 Yi-Shan Road, Shanghai 200233, China

## Abstract

The reference ranges of bone turnover markers (BTMs) were important during the treatment of osteoporosis, and the associations with bone mineral density (BMD) were controversial. The aim of this study was to establish the reference ranges of N-terminal procollagen of type l collagen (P1NP), osteocalcin (OC), and beta C-terminal cross-linked telopeptides of type I collagen (**β**-CTX) in Shanghai area and to investigate the relationships between BTMs and BMD in postmenopausal women. 2,799 subjects recruited in Shanghai City were measured BTMs to establish the reference ranges. Additional 520 healthy postmenopausal women were also measured BTMs, these women measured BMD in addition. BTMs were measured using the Roche electrochemiluminescence system. We used the age range of 35 to 45-year-olds to calculate reference intervals. The reference range of OC was 4.91 to 13.90 ng/mL for women and 5.58 to 16.57 ng/mL for men, P1NP was 13.72 to 32.90 ng/mL for women and 16.89 to 42.43 ng/mL for men, and **β**-CTX was 0.112 to 0.210 ng/mL for women and 0.100 to 0.378 ng/mL for men. BTMs significantly negatively correlated with lumbar spine and femoral and total hip in postmenopausal women (Beta_std_ = −0.157 *~* −0.217, *P* < 0.001). We established the normal reference ranges of P1NP, OC, and **β**-CTX in the Shanghai area. This study also found that BTMs correlated with BMD and suggested that BTMs were the key determining factors of early BMD decreases.

## 1. Introduction 

Bone mineral density (BMD) is used to diagnose osteoporosis and to predict the risk of osteoporotic fracture in postmenopausal women [1, 2]; however, the limitations of BMD measurements in assessing fracture risk have been documented, specifically that less than half of those who suffer a fracture have BMD values in the osteoporotic range [2, 3]. van Coeverden et al., as well as other researchers, confirmed that bone turnover markers (BTMs) had an important impact on bone mass, height, and other growth factors after the onset of puberty in boys and girls [4–6]. A recent study demonstrated that BTMs were useful in fracture prediction and in monitoring and evaluating drug intervention during clinical treatment and that they reflected the tested individual's bone turnover rate in clinical trials [1, 7]. In addition, many studies have found that BTMs can predict the risk of vertebral and hip fractures [8–10] and that they can predict the risk of hip fracture independent of hip BMD [9]. 

BTMs include several enzymes and their decomposition products that come from bone cells and bone matrix components. Among BTMs that reflect the activity of osteoblast cells are serum osteocalcin (OC) in form of N-terminal midmolecule fragment (N-MID) and undercarboxylated osteocalcin (UcOC), N-terminal procollagen of type l collagen (P1NP), and C-terminal procollagen of type l collagen (P1CP). The P1NP that is by the N-terminal enzymatic hydrolysis and is a specific marker that reflected in osteoblasts activity. Its conversion rate is also higher than the soft tissue sources, so the determination of the P1NP can reflect bone formation. BTMs that reflect the activity of osteoclast cells include cross-linked N-telopeptide of type l collagen (NTX), cross-linked C-telopeptide of type l collagen (CTX), tartrate-resistant acid phosphatase (TRAP), and pyridinoline (Pyd). Because it is not being degraded in the blood and excreted by the kidneys, CTXs directly reflect bone matrix collagen degradation. During mature collagen degradation, the C-terminal peptide *α* aspartic acid is converted to *β* aspartic acid. So detection of *β*-CTX were reflected the state of bone resorption.

Most reference ranges have been provided by commercial laboratory kits using approximately 100–200 observations [11]. Therefore, it is essential to establish a valid reference range for clinical use. Once reference ranges for BTMs are established, comparisons of BTMs between healthy individuals and osteoporotic patients can be performed. Until now, there was no reference database of BTMs for the Chinese population. Whereas most studies have measured BTMs and BMD, age and other related factors have only been documented in small groups stratified by menopausal status, gender, or puberty stage, but no large-scale reference database has been compiled. One study indicated that there was no significant bone loss in 30- to 45-year-old women who were calcium balanced and of optimal bone health [12]. Therefore, it is important to establish reference intervals stratified by the known determinants of BTMs, such as age, sex, and body mass index (BMI). In this study, we established a reference database of *β*-CTX, P1NP, and OC levels in healthy Chinese men and women in Shanghai and investigated the relationships between BTMs and BMD in postmenopausal women. 

## 2. Materials and Methods

### 2.1. Subjects

In total, 2,799 individuals aged 20–79 yrs (790 men and 2,009 women) were recruited in March 2009 from the local population in communities of Shanghai. The inclusion criteria were as follows: (1) healthy men and women according to the following excluding criteria; (2) blood tests in this study were accorded with the laboratory normal reference range. The following criteria were used to exclude individuals from the study: (1) serious effects from cerebrovascular disease; (2) diabetes mellitus; (3) chronic renal disease and chronic liver disease; (4) evidence of other metabolic or inherited bone diseases; (5) rheumatoid arthritis or collagen disease; (6) recent major gastrointestinal disease; (7) significant disease of any endocrine organ that would affect bone mass; (8) hyperthyroidism; (9) any neurological or musculoskeletal condition; and (10) any form of calcium and vitamin-D therapy in three months or taking anti-osteoporotic drugs (e.g., bisphosphonates, selective estrogen receptor modulators, and calcitonin). Postmenopausal women who had experienced early menopause (before 45 yrs of age) were excluded. This study was approved by the Ethics Committee of the Shanghai Sixth People's Hospital, Shanghai Jiao Tong University. 

In addition, 520 healthy postmenopausal women with normal liver and kidney function were collected in communities of Shanghai. We carried BMD and the following BTMs in 520 postmenopausal women.

Age, body weight, height, and age of menarche and amenorrhea were recorded. All subjects were medically examined and interviewed using a standardized questionnaire to collect information on life style, smoking habits, the level of physical activity during leisure time, and use of vitamins and medications. All healthy subjects included in the present study had (1) normal blood counts and (2) normal results for liver and kidney function tests.

### 2.2. Biochemical Markers

All biochemical markers were measured at the same time point using a single lot of reagents in one batch, following both the manufacturer's protocol and specialized assay laboratory quality control procedures. Fasting blood samples were collected for the measurement of the serum levels of calcium, phosphate, albumin, glucose, insulin, cholesterol, triglycerides (TG), blood urea nitrogen (BUN), creatinine (Cr), alanine aminotransferase (ALT), aspartate transaminase (AST), r-glutamyl transpeptidase (r-GT), alkaline phosphatase (ALP), and B ultrasound of the spleen, kidneys, hepatobiliary system, and pancreas. 

Serum 25-hydroxyvitamin D [25(OH)D], PTH, *β*-CTX, OC in form of N-MID, and P1NP were measured using an automated Roche electro-chemiluminescence system (Roche Diagnostic Gmbh). The intra- and inter-assay coefficients of variation (CVs) for 25(OH)D were 5.6% and 8.0%, respectively. The lower limit of detection for 25(OH)D was 4 ng/mL (10 mmol/L). The intra- and interassay CVs were 1.4% and 2.9% for PTH, 2.5% and 3.5% for *β*-CTX, 2.9% and 4.0% for OC, and 2.3% and 2.8% for P1NP, respectively.

### 2.3. BMD Measurement

520 postmenopausal women's BMD (g/cm^2^) of the left proximal femur including the total hip, the femoral neck, the trochanter, and Ward's triangle was measured using dual-energy X-ray absorptiometry (DXA) on a Lunar Prodigy GE densitometer (Lunar Corp, Madison, WI, USA). Both of the scanners were calibrated daily, and the coefficient of variability (CV) values for the DXA measurements at L1-4, the total hip, and the femoral neck were 1.39%, 0.7%, and 2.22%, respectively, for the Lunar Prodigy. The long-term reproducibility of the DXA data during the trial, which was based on phantom measurements that were repeated weekly, was 0.45%.

### 2.4. Statistical Analyses

The anthropometric characteristics were presented as the means ± SD, and BTMs were presented as geometric means with 95% confidence intervals (95% CI). Data were analyzed using SPSS 11.0 (SPSS Inc., Chicago, IL, USA). The BTMs ALP, OC, P1NP, PTH, and *β*-CTX were determined to be not normally distributed by the Kolmogorov-Smirnov test. The 95% reference interval for each BTM examined was calculated as the mean ± 1.96 SD. The CI for the lower and upper bounds of the reference intervals was computed as the boundary ± 1.96 standard error (SE). We performed multiple regression analysis between BTMs and BMD, controlling age, height, weight, and years since menopause (YSM).

## 3. Results 

### 3.1. Subject Characteristics

A total of 2,799 subjects were screened for entry into the study by measuring indicators of liver and kidney function and blood calcium and phosphorus: 148 failed the initial screening, leaving 2,651 subjects. 25(OH)D concentrations were divided into four subgroups according to the following criteria: severely deficient (<10 ng/mL), deficient (10–20 ng/mL), insufficient (20-30 ng/mL), and sufficient (≥30 ng/mL). The prevalence of vitamin D insufficiency was 84% in males and 89% in females. The prevalence of vitamin D deficiency was 30% in males and 46% in females. Based on 25(OH)D concentrations, we excluded the subject whose 25(OH)D concentrations were <10 ng/mL in 2651 cases, leaving 705 males and 1836 females last. 

### 3.2. Reference Intervals

Recent studies containing data from larger cohorts of healthy premenopausal women have reported reference intervals for which the age range was 30–45 yr [13–15]. Many factors affect bone turnover markers, including age, weight, and gender. Therefore, we used the age range of 35- to 45-year-olds year to minimize the effects of such factors associated with preanalytical variability, and using this cutoff, we were left with 406 women and 226 men from which to calculate reference intervals. 25(OH)D concentrations of these subjects were 10 ng/mL and more. The anthropometric characteristics of this group of subjects are presented in Table 1.  Table 2 reports the reference intervals for the BTMs studied and includes the medians, geometric means, and 95% reference intervals. The lower half of the reference range (values falling between the lower limit of the reference interval and the median) has been proposed as a target for treatment [7]. This range for OC was 4.91 to 13.90 ng/mL in women and 5.58 to 16.57 ng/mL in men; for P1NP, it was 13.72 to 32.90 ng/mL in women and 16.89 to 42.43 ng/mL in men; for *β*-CTX, it was 0.112 to 0.210 ng/mL in women and 0.100 to 0.378 ng/mL in men. The geometric means and 95% confidence intervals (95% CI) for age-specific BTM values in healthy women and men are shown in Tables 3 and 4.

### 3.3. Effect of Age on Biochemical BTMs

Table 3 shows the geometric means and SDs for the biochemical BTMs in the Chinese women stratified by 5 yr increments. Age-related changes in all biochemical BTMs were calculated by comparison with the BTM values for the reference intervals group. The bone formation markers OC and P1NP decreased with increasing age until the age of 44 and then abruptly increased for women aged 50–59 yr. The bone resorption marker *β*-CTX also decreased with increasing age until the age of 44 before sharply increasing in women > 50 yrs old. All BTMs began to decrease in women aged 70–74 yr.

The geometric means and 95% confidence intervals (95% CI) of the age-specific BTMs in healthy men are shown in Table 4. Age-related changes in all biochemical BTMs were compared with the BTM values of the reference intervals group. The bone formation markers OC and P1NP decreased with increasing age until the age of 59 and then quickly increased in men aged 60–69 yr. The bone resorption marker *β*-CTX also decreased with increasing age until the age of 54, at which point it sharply increased in men > 60 yrs old.

### 3.4. Correlations between BTMs and BMD

In 520 postmenopausal women correlation between BTMs and BMD is given in Table 5. All markers correlated with BMD in lumbar spine, femoral neck, and total hip (Beta_std_ = −0.155 ~ −0.220, *P* < 0.001). When adjusted for age, height, body weight, and YSM, all the three OC, *β*-CTX, and P1NP also correlated with BMD (Beta_std_ = −0.157 ~  −0.217, *P* < 0.001). 

## 4. Discussion 

It is important to establish reference intervals for BTMs from a sample of the healthy young population to use such markers to correctly assess bone turnover in subjects of various ages. In this study, we chose women aged 35–45 yrs for reference interval calculations because these women have achieved peak bone mass and are not yet perimenopausal. Women under 30 yrs of age were excluded to reduce the chance of including women with elevated bone turnover due to skeletal immaturity. In addition, women over the age of 45 were excluded because several studies have demonstrated that BTMs are increased in perimenopausal women because of the estrogen deficiency that occurs after spontaneous menopause in an increase in bone remodeling [16, 17]. It has been recommended that normal reference ranges of BTMs be established in large cohorts (approximately 100–200 subjects) of healthy 35- to 45-year-old premenopausal women with normal BMD [11]. The present study has provided the reference intervals of BTMs were in Chinese men; there are no previous studies of these markers in healthy men, and an age range for reference intervals has not been established. According to Tables 3 and 4, which presents the means for the BTMs within each 5 yr age group, we found that BTMs were stable in the group aged 35 to 45 yr. 

In the present study, the age-related analysis of all subjects revealed a significant negative correlation between each of the bone turnover markers and age. In women, the bone formation markers OC and P1NP decreased with increasing age until the age of 44 before sharply increasing in the 50- to 59-year-old age group. The bone resorption marker *β*-CTX also decreased with increasing age until the age of 44 and abruptly increased in women > 50 yrs old. We found that women aged 30 to 34 yrs had higher levels of *β*-CTX, OC, and P1NP, which may suggest that they had not yet reached skeletal maturity; therefore, we excluded this age group when establishing the reference intervals. Previous studies have found that bone turnover markers are negatively associated with age [10, 18]; however, these particular studies analyzed women aged 20 to 50 yrs and 20 to 40 yrs and therefore did not include postmenopausal women or elderly women. Glover et al. [19] reported that the median values of serum P1NP and *β*-CTX were lower than those reported as reference values in European premenopausal women. The measured reference values for P1NP in the present study were similar to those reported by de Papp et al. [14] in American premenopausal women and by Adami et al. [20] in Italian premenopausal women aged 35 to 45 yrs. In contrast, the reference values for *β*-CTX in our study were lower than those reported for American and Italian premenopausal women [14, 20]. Wu et al. [21] also examined serum *β*-CTX, serum bone ALP, and urinary BTMs in 289 premenopausal Chinese women aged 30–49 yrs; the reported means were 5.18 ng/mL for OC, 17.8 U/L for bone ALP, and 0.250 ng/mL for *β*-CTX [21]. Compared with Wu et al., we measured higher levels of OC. As measured in our current study, the OC levels in premenopausal (17.01 ng/mL) and postmenopausal women (24.34 ng/mL) were remarkably higher than those reported by Wu et al. (4.32 *μ*g/L and 9.14 *μ*g/L, resp.), whose study included 555 women aged 35 to 60 yrs [22]. The different results between the two studies were perhaps due to the season during which they collected blood (May to August). The P1NP level in the postmenopausal women of our population was close to that in a previous study (52.0 (10.5–492.7) ng/mL) in Beijing that included 1,724 postmenopausal women (aged 47 to 108 yr) [23]. However, the serum *β*-CTX level measured in our study was higher than that in the same previous study (0.407 (0.036–2.140) ng/mL) [23]. The dissimilar results from within the same country may be due to differences in the age range of the subjects used to calculate reference intervals, smoking habits, physical activities, or other factors affecting BTMs.

In the cohort of men we studied, age-related changes in BTMs were discovered. The bone formation markers OC and P1NP decreased with increasing age until the age of 59 and then abruptly increased in the 60- to 69-year-old group. The bone resorption marker *β*-CTX also decreased with increasing age until the age of 54 and then sharply increased in men > 60 yrs old. Elevated levels of bone markers in young men reflect the active bone turnover that occurs during the formation of peak BMD. We found that the age at which BTMs decreased in men was about 10 yrs later than in women. This finding is consistent with the fact that the age of onset of osteoporosis in men is 10 yrs later than in women, perhaps because women finish growing and achieve peak BMD earlier than men [24]. The results of a study by Szulc et al. were similar to ours: before 25 yrs of age, markers of bone formation (OC, BAP, and P1NP) were high, but they decreased and reached their lowest levels at the age of 56–60 yrs [25]. To date, most studies have found a decrease in BTMs in men until the age of 50–60 yrs [26]. However, the number of young men studied was small and the age range investigated began after peak values were achieved [27]. 

Previous reports have indicated that 25(OH)D levels are negatively correlated with serum PTH, OC, P1NP, and *β*-CTX in women and men [28, 29]. These reports noted that patients with 25(OH)D insufficiency exhibit higher levels of bone turnover than those with sufficient 25(OH)D levels [29, 30]. However, most studies were carried out in elderly osteoporotic women or less cared for young men and women. Vitamin D insufficiency is associated with high levels of BTMs (including OC, *β*-CTX, P1NP, and PTH) in young individuals, so we also examined the 25(OH)D levels in our 35- to 45-year-old subjects to avoid its effect on BTMs. We found that the level of 25(OH)D was 20.58 ng/mL in women aged 35–45 yrs and 22.89 ng/mL in men of the same age. No differences were found in 25(OH)D levels between the two groups. The levels of 25(OH)D in 35- to 45-yr-old subjects reflected vitamin D status in healthy Chinese adults. 

The study found that OC, *β*-CTX, and P1NP and the BMD of lumbar spine, femoral neck, and total hip were significantly negatively correlated. The effects remained after being adjusted for age, height, weight, and YSM. After menopause, an increased bone turnover is related to bone loss by an imbalance between bone formation and bone resorption. A number of studies have found significant relationship between BTMs and bone mass [31, 32]. In contrast, Kawana et al. [33] failed to report significant correlations between ultrasound parameters and biochemical markers of bone remodeling (osteocalcin, Pyr, and D-Pyr) in postmenopausal women aged 48–57 years. Several studies have indicated a potential relationship between BTMs and QUS in postmenopausal women [34]. This means that the combination of bone resorption and bone formation markers is a good predictor for loss of bone mass.

Our study has the following strengths: (1) the sample size was large enough (greater than 600 men and 1,000 women) to avoid sample error; (2) the random selection of women from the local population, thereby covering a wide age-range and being representative of the Chinese population; and (3) the participants were well characterized and the exclusion criteria were detailed and evidence based to ensure a more precisely characterized sample population. Thus, numerous factors to minimize biological variability were considered and controlled. 

Certain limitations of this study must also be acknowledged. First, we did not measure BMD in 2,799 subjects (790 males and 2,009 females). Second, the purpose of this study was to establish the reference intervals for BTMs, for which we analyzed a sufficient number of young men and women; however, compared with the number of young subjects, the sample size of elderly subjects was not adequate, particularly when examining elderly men. Finally, the cross-sectional nature of the study design is a limitation.

## 5. Conclusion 

The present study provides reference interval values for BTMs in healthy men and women. These reference ranges are based on data showing that BTMs are stable between the ages of 35 and 45 yrs. These results will contribute to the appropriate assessment of bone turnover in Shanghai area population and offer a comparison to measurements obtained in other populations. This study also found that BTMs correlated with BMD in Chinese postmenopausal women and suggested that BTMs were the key determining factors of early BMD decreases.

## Figures and Tables

**Table 1:** Anthropometric characteristics and other variables in the men and women aged 35–45 yr used to calculate the reference intervals of the bone turnover markers.

Characteristics	Men (*n* = 226)	Women (*n* = 406)
Age (year)	39.8 ± 3.2 (35–45)	39.4 ± 3.2 (35–45)
Height (cm)	173.2 ± 5.9 (165.8–180.1)	162.0 ± 4.9 (153.7–172.5)
Weight (kg)	75.0 ± 12.5 (61.6–85.5)	58.3 ± 7.1 (44.7–68.3)
BMI (kg/m^2^)	25.4 ± 3.1 (20.8–26.4)	22.2 ± 2.6 (17.9–24.6)

*Data are presented as the mean ± SD. Parenthesis for minimum and maximum.

**Table 2:** Medians, geometric means, and 95% reference intervals for the bone turnover markers in 35–45-year-old subjects.

Measurement	Women (*n* = 406)			Men (*n* = 226)		
	Median	Geometric mean	95% reference interval	Median	Geometric mean	95% reference interval
ALP (U/L)	53.00	54.80	29.55–82.04	63.00	63.87	35.52–94.16
OC (ng/mL)	13.90	15.05	4.91–22.31	16.57	18.01	5.58–28.62
P1NP (ng/mL)	32.90	35.22	13.72–58.67	42.43	44.01	16.89–65.49
*β*-CTX (ng/mL)	0.210	0.241	0.112–0.497	0.378	0.400	0.100–0.612
25(OH)D (ng/mL)	20.58	20.98	10.97–32.15	22.89	23.55	11.11–34.43
PTH (pg/mL)	35.02	37.30	15.52–66.78	30.39	33.92	14.61–63.22

Values were obtained from 35–45-year-old healthy men and women.

**Table 3:** Geometric means and 95% confidence intervals (95% CI) for the age-specific measurements of bone turnover markers in healthy women.

Age	Number	*β*–CTX (ng/mL)	P1NP (ng/mL)	OC (ng/mL)	PTH (pg/mL)
20–24	145	0.412* (0.354–0.470)	54.97* (47.87–62.06)	21.23* (18.72–22.47)	37.20 (35.07–39.33)
25–29	386	0.335* (0.304–0.367)	45.94* (41.38–50.49)	18.48* (17.19–19.77)	35.70 (34.29–37.10)
30–34	247	0.280* (0.230–0.313)	40.21* (35.78–44.63)	16.80* (15.33–18.26)	36.45 (34.76–38.13)
35–39	210	0.250 (0.221–0.284)	35.93 (31.72–40.13)	15.37 (13.95–16.78)	38.10 (35.99–40.21)
40–44	178	0.239 (0.201–0.271)	34.27 (30.50–38.04)	13.84 (12.48–15.27)	38.55 (36.17–40.94)
45–49	104	0.278* (0.221–0.310)	36.35* (31.10–41.58)	14.84* (12.88–16.79)	38.99 (36.09–41.89)
50–54	138	0.470* (0.399–0.541)	52.72*(45.54–56.90)	23.46* (20.44–26.46)	35.83 (33.58–38.08)
55–59	184	0.474* (0.407–0.540)	50.21* (43.85–56.57)	24.94* (22.19–27.68)	36.50 (34.76–38.24)
60–64	51	0.441* (0.307–0.584)	47.07* (34.78–59.36)	26.40* (20.28–32.60)	37.92 (32.71–43.13)
65–69	70	0.471* (0.356–0.585)	50.61* (38.11–63.11)	25.57* (22.90–28.22)	36.13 (32.72–39.55)
70–74	77	0.431* (0.297–0.565)	50.04* (37.94–62.13)	26.50* (20.82–32.17)	35.57 (30.94–38.21)
75–79	66	0.384* (0.284–0.482)	45.44* (35.60–54.47)	24.11* (18.85–29.37)	36.25 (29.70–38.81)
Reference value^a^	406	0.242 (0.218–0.297)	35.22 (30.67–40.10)	15.05 (13.81–16.59)	37.30 (33.18–40.92)

All values are presented as the geometric means (95% CI).

*P < 0.05 versus reference values.

^a^Reference values were calculated from the 35–45-year-old cohort as the geometric mean (95% CI).

**Table 4:** Geometric means and (95% CI) for the age-specific measurements of bone turnover markers in healthy men.

Age	Number	*β*-CTX (ng/mL)	P1NP (ng/mL)	OC (ng/mL)	PTH (pg/mL)
20–24	43	0.745* (0.464–1.024)	64.44* (41.81–87.05)	25.21* (17.57–28.86)	31.77 (25.61–39.94)
25–29	72	0.504* (0.415–0.551)	51.81* (43.42–60.20)	21.08* (18.12–24.06)	34.73 (31.14–38.33)
30–34	93	0.419* (0.353–0.482)	44.68* (38.25–51.10)	18.51* (15.97–21.05)	34.06 (30.46–37.67)
35–39	102	0.402 (0.333–0.470)	44.22 (37.64–57.01)	18.34 (15.66–21.02)	35.51 (32.40–38.62)
40–44	117	0.402 (0.318–0.485)	43.98 (36.78–51.78)	17.85 (14.89–20.79)	35.09 (31.89–38.29)
45–49	45	0.340* (0.252–0.428)	36.62* (29.37–43.88)	16.02* (11.97–20.05)	30.01 (25.92–34.20)
50–54	53	0.342* (0.267–0.414)	39.59* (32.23–46.93)	16.69* (13.72–19.65)	34.60 (30.86–38.35)
55–59	64	0.353* (0.278–0.427)	36.93* (30.62–43.23)	15.58* (13.07–18.09)	35.80 (31.57–40.02)
60–64	45	0.393* (0.276–0.508)	41.19* (31.63–50.13)	20.95* (16.32–25.66)	36.94* (31.27–42.60)
65–69	26	0.352* (0.218–0.485)	39.39* (27.48–50.87)	19.43* (12.28–25.51)	37.44* (30.58–44.31)
70–74	21	0.383* (0.167–0.598)	40.00* (26.65–53.33)	21.84* (15.95–27.73)	37.45* (30.52–44.38)
75–79	24	0.355* (0.208–0.501)	40.10* (25.60–54.69)	20.86* (14.72–27.00)	36.34* (29.98–42.70)
Reference value^a^	226	0.400 (0.310–0.479)	44.01 (37.27–52.97)	18.01 (14.91–20.97)	33.92 (30.21–38.17)

All values are presented as the geometric means (95% CI).

*P < 0.05 versus reference values.

^a^Reference values were calculated from the 35–45-year-old cohort as the geometric mean (95% CI).

**Table 5:** Standardized correlation coefficients (Beta_std_) between BTMS and BMD in 520 postmenopausal women.

BTMs	L1-4 BMD		Femoral neck BMD		Total hip BMD	
	Not adjusted	Adjusted	Not adjusted	Adjusted	Not adjusted	Adjusted
*β*-CTX	−0.155**	−0.157**	−0.188**	−0.182**	−0.207**	−0.195**
P1NP	−0.197**	−0.201**	−0.169**	−0.193**	−0.220**	−0.217**
OC	−0.191**	−0.188**	−0.178**	−0.176**	−0.209**	−0.199**

Data adjusted age, height, body weight, and YSM are given.

**P < 0.01.
